# Association between testosterone and serum soluble α-klotho in U.S. males: a cross-sectional study

**DOI:** 10.1186/s12877-022-03265-3

**Published:** 2022-07-11

**Authors:** Zilong Zhang, Shi Qiu, Xinyi Huang, Kun Jin, Xianghong Zhou, Tianhai Lin, Xiaoli Zou, Qiuxiang Yang, Lu Yang, Qiang Wei

**Affiliations:** 1 Department of Urology and Institute of Urology and National Clinical Research Center for Geriatrics, West China Hospital, Sichuan University, No. 37, Guoxue Alley, Chengdu, 610041 Sichuan China; 2 West China School of Public Health and West China Fourth Hospital, Sichuan University, Chengdu, China

**Keywords:** Sex hormones, Soluble α-Klotho, NHANES

## Abstract

**Purpose:**

Testosterone plays a crucial role in males, and the deficiency of testosterone leads to multiple adverse health conditions. Klotho is a recently discovered protein encoded by antiaging gene *klotho*. Both the levels of testosterone and klotho change with aging, so the relationship between them is worth exploring. The purpose of this study was to investigate whether total testosterone is associated with serum klotho levels in U.S. males aged 40–79 years.

**Methods:**

Included in this study were 3750 male participants from the 2011 to 2016 National Health and Nutrition Examination Survey, aged 40–79 years with included information on klotho and sex hormones. The sex steroid hormone levels and klotho concentrations were assayed in laboratories using the recommended methods according to Nutrition Examination Survey guidelines. The association between sex hormones and klotho was calculated using multivariate linear regression models after adjustment for several possible confounding variables.

**Results:**

Among the 3750 participants, the total testosterone concentration was 399.048 ± 184.780 ng/dL, and the testosterone deficiency prevalence was 1160 (30.942%). The geometric mean of serum klotho levels was 791.000 pg/mL. In the adjusted models, klotho increased 0.165 pg/mL for every 1 ng/dL increase of total testosterone (*p =* 0.004). In addition, estradiol (β 2.232; 95% CI 0.588–3.876; *p =* 0.032) and sex hormone-binding globulin (β 2.013; 95% CI 1.173–2.583; *p =* 0.002) were also positively associated with klotho concentrations.

**Conclusion:**

This study reported a significant association between klotho and sex hormones in the U.S. male population. The levels of klotho in men increased with total testosterone, estradiol and sex hormone-binding globulin levels, which may have implications for future research and clinical practice.

**Supplementary Information:**

The online version contains supplementary material available at 10.1186/s12877-022-03265-3.

## Introduction

Klotho is a single-pass transmembrane protein encoded by the aging–suppressor gene *klotho* [1]. Klotho contains a short intercellular domain and two long extracellular domains with a high sequence similarity [2, 3]. The cleaved extracellular domains by ADAM10 and ADAM17 can be released and identified as soluble Klotho (S-Klotho), which can be used to reflect renal klotho expression [4]. Three coding products have been identified: α-Klotho, β-Klotho, and γ-Klotho. α-Klotho acts as a coreceptor of FGF23, which is mainly expressed in the distal convoluted tubules mostly, as well as the choroid plexus and the parathyroid glands [5, 6]. Previous experiments have found that klotho expression in aging mice is physiologically downregulated [1]. Additionally, it has been reported that a deficiency of α-Klotho has pathophysiological effects on multiple aging-related disorders such as cardiovascular disease [7, 8], cancer [9], type 2 diabetes [10], and chronic kidney disease [11]. A strong relationship between klotho and all-cause mortality has also been reported [12].

Testosterone is a steroid hormone produced predominantly in the Leydig cells of the testis; ovaries and adrenal glands can also produce testosterone in small quantities [13, 14]. Thus, this explains the significantly higher concentration of testosterone in males than in females. The production and release of testosterone are regulated by the hypothalamic-pituitary–gonadal axis [15]. Testosterone plays essential roles in muscle growth and the development of secondary sexual characteristics in males [13]. Testosterone levels physiologically decreased with aging [16]. Testosterone deficiency affects about 7% man in their 50 s, and can lead to higher risk of diseases related to aging including cardiovascular disease, diabetes, low lean mass, and metabolic syndrome [16–20].

Numerous studies have demonstrated associations between hormones and klotho. Soluble α-klotho is positively associated with parathyroid hormone levels in men and negatively in women [21]. Klotho may also mediate the abnormal secretion of parathyroid hormone in patients with hyperparathyroidism [22]. However, since both testosterone and klotho change with age, the relationship between them is worth further investigating. One previous study demonstrated that testosterone can upregulate the expression of the *klotho* gene, and an additional study with a specific cohort revealed a positive relationship between testosterone and S-klotho in healthy sedentary middle-aged adults [23, 24]. Another recent study assessed the relationship between testosterone levels and klotho in young males and also showed a positive correlation [25]. To the best of our knowledge, no research has been conducted to study the relationship between testosterone and S-klotho levels in males using a nationally representative population. Thus, the primary aim of this present study is to investigate the association between testosterone and S-klotho concentrations in males using the National Health and Nutrition Examination Survey (NHANES). Previous studies also indicated that a relationship between estradiol and Klotho may exist [26]. In the present study, estradiol (E2) and sex hormone-binding globulin (SHBG) are also analyzed as independent variables, due to their physiological relationship with testosterone [27, 28].

## Methods

### Population and study design

The National Center for Health Statistics (NCHS) conducted the NHANES, which is a cross-sectional study aiming to investigate the overall status of health and nutrition among a nationally representative U.S. population. The survey is processed as a two-year cycle, including sections for demographics, dietary information, medical examination, laboratory values, and the questionnaire. The NCHS Ethics Review Board approved the NHANES study, and written informed consents were signed by all participants. All detailed information and raw data of the NHANES is publicly available at https://www.cdc.gov/nchs/nhanes/.

**Fig. 1:**
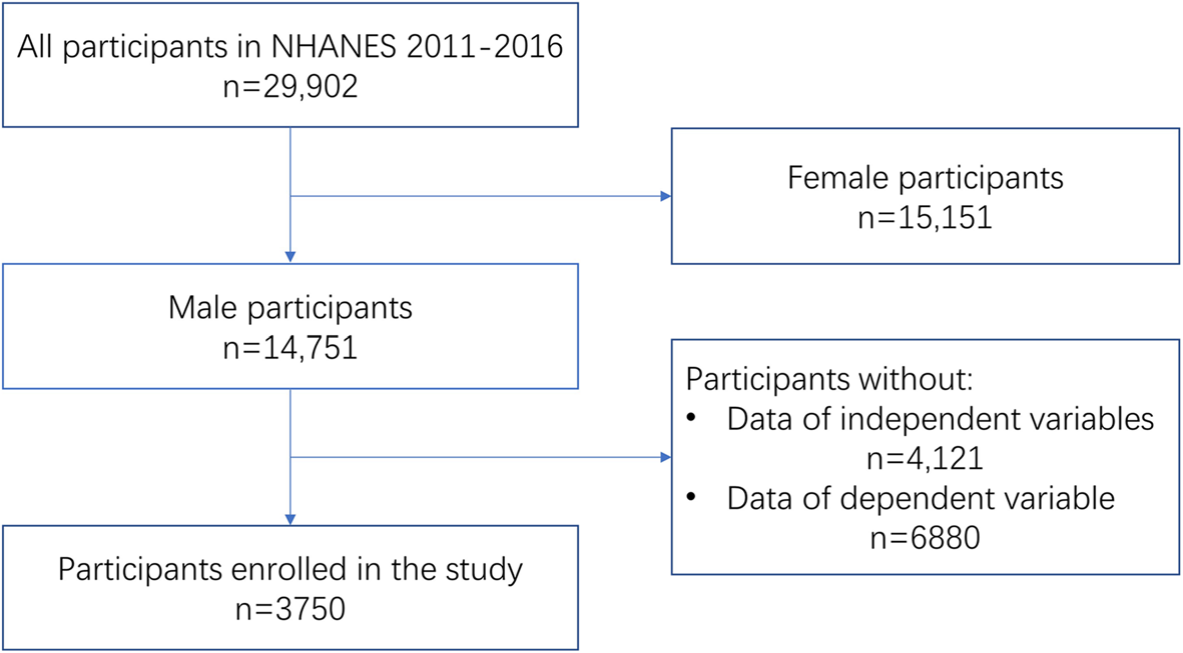
The flowchart of participants selection

The independent variables are total testosterone (TT), E2, SHBG, ratio of testosterone to E2, and testosterone deficiency (TD). The dependent variable is S-klotho levels. The S-klotho concentrations were measured during the period of 2007–2016. The data for testosterone was available in 2011–2016, while estradiol and SHBG data were available in 2013–2016. Thus, the present study used data restricted to the 3 continuous cycles from 2011 to 2016. All included participants were males between the ages of 40–79 years, and those without reported information for the independent or dependent variables were excluded. A total of 3,750 participants were enrolled in this study. The flowchart of participants selection is presented in Fig. 1.

### Measurement of independent variables

TT, E2, and SHBG were measured for all participants aged six years or older who did not meet the exclusion criteria. The serum samples were stored at -20 °C. Details about sample collection and storage are discussed in the NHANES Laboratory/Medical Technologists Procedures Manual. TT and E2 were measured using the validated isotope dilution liquid chromatography tandem mass spectrometry (ID-LC–MS/MS) method according to guidelines set by the National Institute for Standards and Technology. The samples were measured at the same time in the morning, afternoon, and evening. The steroids were isolated from serum samples by liquid–liquid extraction, and stable isolated testosterone and E2 were used as internal standards. The measurement of SHBG was performed using the chemo-luminescence method on the products of reaction between SHBG and its immune-antibodies. The microparticles of the products were captured by the electrode, and a chemiluminescent reaction occurred. The results were measured and read by a photomultiplier tube. The ratio of testosterone to E2 was calculated after transferring the values into the same unit of measurement. According to the American Urological Association Guidelines, TD is defined as a condition with TT less than 300 ng/dL [29]. The lower limit of detection (LLOD) for TT, E2, and SHBG are 0.75 ng/mL, 2.994 pg/mL, and 0.800 nmol/L, respectively. More information on this laboratory method is discussed at https://wwwn.cdc.gov/Nchs/Nhanes/2013-2014/TST_H.htm.

### Measurement of dependent variable

Blood samples were collected in mobile examination centers on dry ice from all participants who agreed to use their serum samples for the study and signed the informed consent. The samples were transferred and stored at -80 °C. Analyses were performed with the IBL ELISA method, a commercially available ELISA kit produced by IBL International, Japan. In order to validate the performance of the kits, the IBL ELISA method was used to measure the S-klotho concentrations of human samples at the start of the study. All samples were bisected and measured separately, and the average of the two values was utilized as the final result. The assay sensitivity was 6 pg/mL. Details about the laboratory methodology, as well as quality assurance and monitoring, are available at https://wwwn.cdc.gov/Nchs/Nhanes/2007-2008/SSKL_E.htm.

### Measurement of covariates

According to a previous study, the analyses on association between sex hormones and S-klotho were adjusted for several possible confounding variables. Demographics variables include age (continuous), race (categorical: Mexican American, Other Hispanic, Non–Hispanic White, Non–Hispanic Black, and Others), education level (categorical: less than high school, high school or general educational development, and above high school), marital status (categorical: married or living with partner, and living alone), and ratio of income-poverty (categorical: ≦1.3 as low, 1.3–3.5 as middle, and > 3.5 as high). The examination variables were body mass index (BMI) (categorical: ≦25 as normal weight, 25–30 as overweight, and > 30 as obese), which was calculated by dividing weight by height squared, and the time of venipuncture (categorical: morning, afternoon, and evening). The questionnaire variables included smoking status (categorical: less than 100 in entire life represented never, 100 or more but no smoking now represented former, and 100 or more while smoking now represented current), alcohol consumption (categorical: 0 g/day as none, 0.1–27.9 g/day for men and 0.1–13.9 g/day for women as moderate, and 28.0 g or more/day for men and 14.0 g or more/day for women as heavy), physical activity (categorical: less than moderate, moderate, and vigorous), and cardiovascular disease (CAD) score (categorical: 0 as none, 1 as slight, and 2–5 as heavy). The scores were generated by accumulated points according to the following guidelines. Participants got one point each for hypertension, history of transient ischemic attack or stroke, and coronary artery disease. One point was added if they had impaired glucose tolerance, and two points for the diagnosis of diabetes. A categorical variable was defined to assess physical activity intensity: less than moderate as 0 min/week of moderate-to-vigorous physical activity, moderate as between 0 and 150 min/week, and vigorous as more than 150 min/week (according to the national physical activity guidelines).

### Statistical analysis

In the present study, data were presented as mean ± standard deviation for continuous variables, and numbers (proportion) for categorical variables. S-klotho concentration was presented in geometric mean (GM), median, and upper and lower quartiles due to its highly skewed distribution. The Kruskal–Wallis H test was applied for continuous variables and a chi-square test was applied for categorical variables, in order to calculate if there were any differences of characteristics among participants in different testosterone tertiles. Differences of sex hormones and klotho among different age tertiles were also analyzed. Multivariate linear regression models were used respectively when evaluating the associations between TT, E2, SHBG, or the ratio of testosterone to E2 and S-klotho, and a multivariate binomial logistic regression model was applied when analyzing the association between TD and S-klotho. Covariates included age, race, family income-poverty ratio, education level, marital status, BMI, CAD score, time of venipuncture, smoking, drinking, and physical activity. Both unadjusted and adjusted models for each pair of independent and dependent variable were presented in the results. Subgroup analyses were performed with interaction terms on possible confounding covariates including age [30], BMI [31], and physical activity [32], and the log likelihood ratio test was performed. Considering the complex study design of NHANES and in order to make the study population nationally representative, a sample weight was put on each participant. In accordance with the NHANES Survey Methods and Analytic Guidelines, recommended weighting methodology was applied during the analysis process. For analytic results below the LLOD, the value of LLOD divided by the square root of two was placed. Missing values of covariates were replaced by the median for the continuous variables, and the mode for categorical variables. All analyses were conducted by the software package R (http://www.R-project.org, The R Foundation) and EmpowerStats (www.empowerstats.com, X&Y Solution, Inc). The *p*-values of multiple testing were all adjusted and two-tailed *p <* 0.05 was considered statistically significant.

## Results

A total of 29,902 participants were enrolled in the NHANES study from 2011 to 2016. Among all participants, there were 15,151 females and 14,751 males. 4121 men with missing independent variable values and 6880 men without data on dependent variables were excluded. Finally, a total of 3750 participants were selected for the present study. The flowchart of participant selection is shown in Fig. 1.

Table 1 presents the baseline characteristics of both the total population and the participants divided into TT tertiles. The TT concentration among all participants was 399.048 ± 184.780 ng/dL, and the TD prevalence was 1160 (30.942%). GM of S-klotho level was 791.000, which was observed at a significantly higher level (*p =* 0.003) in tertle 3 (GM = 811.953) than in tertile 1 (GM = 777.701). E2 and SHBG levels were also generally higher in men with higher testosterone levels (*p* values < 0.001). Compared to men in the lowest tertile, those in the highest tertile of TT were significantly more obese, married, had a higher CAD score, and with physical activity less than moderate.

**Table 1:** Baseline characteristics of male participants in 2011–2016 NHANES^a^

	Total	Tertile 1 of TT	Tertile 2 of TT	Tertile 3 of TT	*p* value ^b^
Numbers of subjects	3750	1249	1250	1250	-
Age, year	57.961 ± 10.701	58.283 ± 10.926	57.926 ± 10.814	57.670 ± 10.356	0.357
Race					0.167
Mexican American	545 (14.533%)	181 (14.492%)	191 (15.280%)	173 (13.840%)	
Other Hispanic	413 (11.013%)	134 (10.729%)	138 (11.040%)	141 (11.280%)	
Non-Hispanic White	1459 (38.907%)	499 (39.952%)	452 (36.160%)	507 (40.560%)	
Non-Hispanic Black	785 (20.933%)	250 (20.016%)	264 (21.120%)	271 (21.680%)	
Others	548 (14.613%)	185 (14.812%)	205 (16.400%)	158 (12.640%)	
Income-poverty ratio					0.028
Low	1051 (30.455%)	370 (32.118%)	313 (27.265%)	368 (32.000%)	
Middle	1187 (34.396%)	369 (32.031%)	426 (37.108%)	392 (34.087%)	
High	1213 (35.149%)	413 (35.851%)	409 (35.627%)	390 (33.913%)	
Education level					0.004
Less than high school	920 (25.075%)	296 (24.144%)	291 (23.833%)	333 (27.273%)	
High school or GED	792 (21.586%)	266 (21.697%)	237 (19.410%)	288 (23.587%)	
Above high school	1957 (53.339%)	664 (54.160%)	693 (56.757%)	600 (49.140%)	
Marital status					< 0.001
Married/living with partner	2686 (71.627%)	933 (74.700%)	906 (72.480%)	846 (67.680%)	
Living alone	1064 (28.373%)	316 (25.300%)	344 (27.520%)	404 (32.320%)	
BMI					< 0.001
Normal weight	855 (23.083%)	142 (11.564%)	256 (20.712%)	457 (36.885%)	
Over-weight	1516 (40.929%)	447 (36.401%)	544 (44.013%)	525 (42.373%)	
Obese	1333 (35.988%)	639 (52.036%)	436 (35.275%)	257 (20.743%)	
CAD score					< 0.001
None	1555 (43.051%)	417 (35.071%)	518 (42.845%)	620 (51.113%)	
Slight	1018 (28.184%)	324 (27.250%)	350 (28.950%)	344 (28.359%)	
Heavy	1039 (28.765%)	448 (37.679%)	341 (28.205%)	249 (20.528%)	
Time of venipuncture					< 0.001
Morning	1830 (48.800%)	475 (38.030%)	580 (46.400%)	774 (61.920%)	
Afternoon	1398 (37.280%)	548 (43.875%)	485 (38.800%)	365 (29.200%)	
Evening	522 (13.920%)	226 (18.094%)	185 (14.800%)	111 (8.880%)	
Smoking					< 0.001
Never	1593 (42.514%)	523 (41.907%)	562 (44.960%)	507 (40.625%)	
Former	1486 (39.658%)	561 (44.952%)	491 (39.280%)	434 (34.776%)	
Current	668 (17.828%)	164 (13.141%)	197 (15.760%)	307 (24.599%)	
Alcohol drinking					0.001
None	2471 (71.169%)	856 (74.177%)	812 (70.732%)	802 (68.606%)	
Moderate	406 (11.694%)	142 (12.305%)	122 (10.627%)	142 (12.147%)	
Heavy	595 (17.137%)	156 (13.518%)	214 (18.641%)	225 (19.247%)	
Physical activity					0.006
Less than Moderate	2026 (54.099%)	716 (57.464%)	646 (51.721%)	663 (53.082%)	
Moderate	342 (9.132%)	120 (9.631%)	122 (9.768%)	100 (8.006%)	
Vigorous	1377 (36.769%)	410 (32.905%)	481 (38.511%)	486 (38.911%)	
Total testosterone, ng/dL	399.048 ± 184.780	1.800–306.850^c^	307.000–447.160^c^	447.580–2543.990^c^	-
Estradiol, pg/mL	24.571 ± 9.755	20.368 ± 7.959	24.406 ± 8.930	28.710 ± 10.312	< 0.001
SHBG, nmol/L	48.421 ± 25.298	34.494 ± 16.738	43.998 ± 16.706	65.851 ± 28.869	< 0.001
Testosterone/estradiol ratio	0.050 ± 0.022	0.036 ± 0.015	0.048 ± 0.015	0.065 ± 0.023	< 0.001
Testosterone deficiency					-
No	2589 (69.058%)	-	-	-	
Yes	1160 (30.942%)	-	-	-	
S-Klotho, pg/mL^d^	791.000, 787.050 648.700–963.075	777.701, 768.400 633.200–953.800	783.731, 782.200 644.600–948.250	811.953, 808.400 670.625–988.050	0.003

*Abbreviation*: *NHANES* The National Health and Nutrition Examination Survey, *GED* General educational development, *BMI* Body mass index, *CAD score* The cardiovascular disease score, *SHBG* The sex hormone-binding globulin

^a^ Continuous variables are presented as mean ± standard deviation (SD) and categorical variables are presented as numbers (proportion)

^b^ *P* values of differences among different TT tertiles were calculated by Kruscal Whallis H test (continuous variables) or chi-square test (categorical variables)

^c^ Ranges of total testosterone tertiles were presented as Min–Max

^d^ S-Klotho concentration is presented in geometric mean, median, upper and lower quartiles due to its highly skew distribution

Table 2 shows the association between sex hormones and S-Klotho levels among U.S. males. In the fully adjusted models, TT (β 0.165; 95% CI 0.065–0.266; *p =* 0.004), E2 (β 2.232; 95% CI 0.588–3.876; *p =* 0.032), and SHBG (β 2.013; 95% CI 1.173–2.583; *p =* 0.002) were all significantly positively associated with S-klotho concentrations. Males with TD were observed to have about a 18.062 pg/mL lower concentration of klotho than males without TD. However, this result was not statistically significant (*p =* 0.135).

**Table 2:** The association between hormones and S-Klotho concentration among the U.S. males in NHANES 2011–2016

	Number of Subjects	Unadjusted model		Adjusted model^a^	
		β, (95% CI)	*p* values	β, (95% CI)	*p* values
TT	3749	0.133 (0.056, 0.210)	0.001	0.165 (0.065,0.266)	0.004
E2	2526	1.963 (0.388, 3.538)	0.021	2.232 (0.588, 3.876)	0.032
SHBG	2356	1.235 (0.627, 1.843)	< 0.001	2.013 (1.173, 2.583)	0.002
T/E2 ratio	2525	220.419 (-443.164, 884.002)	0.520	455.345 (-398.496, 1309.186)	0.331
TD	3749	-13.794 (-34.003, 6.416)	0.188	-18.062 (-40.968, 4.843)	0.135

*Abbreviation*: *NHANES* The National Health and Nutrition Examination Survey, *CI* Confidence interval, *TT* Total testosterone, *E2* Estradiol, *SHBG* The sex hormone-binding globulin, *T/E2 ratio* The ratio of testosterone to estradiol, *TD* Testosterone deficiency

^a^ The model was fully adjusted by age, race, education level, marital status, family income-poverty ratio, BMI, time of venipuncture, CAD score, smoking status, alcohol consumption, and physical activity

The results of this study also showed that, compared with younger groups, klotho levels in participants aged 63–79 decreased (*p =* 0.032) and SHBG levels increased (*p <* 0.001). However, differences of TT and E2 were not shown with statistical significance (see Additional Table 1).

The positive association between TT and S-Klotho were observed in groups with older age (β 0.129, 95% CI 0.057–0.202, *p <* 0.001), less than moderate physical activity (β 0.107, 95% CI 0.036–0.178, *p =* 0.003), and without normal weight (β 0.105, 95% CI 0.022–0.188, *p =* 0.013 in the overweight group; β 0.104, 95% CI 0.000–0.207, *p =* 0.050 in the obese group). However, no statistically significant results on tests of interactions of subgroup analyses stratified by age, BMI, and physical activity were observed in association between TT and S-klotho (see Additional Table 2, 3, 4 p for interaction equals 0.182, 0.909, and 0.837, respectively).

## Discussion

This cross-sectional study was conducted using a representative sample of 3750 males in the U.S. The results of the current study showed that TT, E2, and SHBG levels were positively associated with S-klotho levels in men aged 40–79 years. The subgroup analyses stratified by age, BMI, and physical activity gave results of no significant interaction terms.

The present study demonstrated that S-klotho increased 0.133 pg/mL for every 1 ng/dL increase in TT among U.S. males. This result was supported by a previous study conducted by Dote-Montero, M. et al. using a sample of 73 healthy sedentary middle-aged participants (34 men), in which the positive association between free testosterone and S-klotho was observed [24]. The possible mechanism was discussed in another study by Hsu, S. C. et al. both in vivo and in vitro. The androgen receptor (AR), a primary mediator of androgen physiological function, was observed to have a functional association with klotho in the distal convoluted tubules. Dihydrotestosterone, produced by the reaction of testosterone with 5α reductase, can bind with AR, and interact with androgen receptor elements directly. The *klotho* gene promoter is subsequently activated, followed by *klotho* mRNA expression [23].

Indicated in this study was also the positive association between E2 and SHBG with S-klotho levels. Similar to testosterone, estrogen regulates the expression of the klotho gene through estrogen receptor α [33]. However, an earlier study reported that expression of the renal *klotho* gene was increased in female mice with estrogen deficiency, and that *klotho* gene expression decreased after supplementation of estrogen [26]. These results seem to contradict current research. A possible explanation is the difference of participant genders between the two studies. Estradiol was primarily produced in testes converted from testosterone by the enzyme aromatase cytochrome P450 protein [28]. Approximately 44%–60% of total serum testosterone was bound with SHBG, the primary binding protein of testosterone. It was speculated that both E2 and SHBG are positively correlated with S-klotho, due to the relationship between TT and S-klotho [27].

Also noteworthy is the effect of age on this relationship. This study found that klotho levels varied among different age groups, while TT did not. TT was significantly associated with klotho in the group older than 60 years, but not in the group younger than 60 years. However, no interaction effect of age was shown in association between TT and klotho. Future studies are necessary to assess this difference of association between testosterone and klotho among age classifications.

There are multiple strengths in this present study. Firstly, this study used a nationally representative population with a relatively large sample size of 3750 males. Secondly, this study considered the differences in the production and levels of testosterone in men and women, in which only males were analyzed. However, several limitations were also present. The primary limitation is that this is a cross-sectional study, from which causality cannot be determined. Another limitation is that participants' plasma testosterone levels were only measured at a single time point. According to AUA guidelines, two levels from different times should be assayed, since plasma testosterone levels in the same individual can change over time. Finally, there may still be potentially confounding factors affecting the association between TT and S-Klotho. This requires future multicenter cohort studies to validate the results of this study by incorporating more potential confounders and having testosterone levels measured according to AUA guidelines. This study provides a potential method for early screening of the aging patients in the context of age-related androgen decline and health deterioration., as well as a potential therapeutic target for treating these patients.

## Conclusions

The current study demonstrated a positive association between TT and S-Klotho. E2 and SHBG were found to be related with serum S-klotho levels. This result validated the applicability of the results by previous studies to the general population, and may provide some inspiration for further research and clinical practice in the future.

## Supplementary Information

**Additional file 1.** Differences of sex hormones and klotho levels among age classifications^a^.

**Additional file 2.** Subgroup analysis of association between sex hormones and S-Klotho stratified by age among the U.S. males in NHANES 2011-2016^a^.

**Additional file 3.** Subgroup analysis of association between sex hormones and S-Klotho stratified by BMI among the U.S. males in NHANES 2011-2016^a^.

**Additional file 4.** Subgroup analysis of association between sex hormones and S-Klotho stratified by physical activity among the U.S. males in NHANES 2011-2016^a^.

## Data Availability

The datasets analyzed during the current study are available in the NHANES repository, https://www.cdc.gov/nchs/nhanes/.

## Abbreviations

S-Klotho: Soluble Klotho
NHANES: The National Health and Nutrition Examination Survey
TT: Total testosterone
E2: Estradiol
SHBG: The sex hormone-binding globulin
TD: Testosterone deficiency
NCHS: The National Center for Health Statistics
ID-LC–MS/MS: Isotope dilution liquid chromatography tandem mass spectrometry
LLE: Liquid–liquid extraction
LLOD: The lower limit of detection
BMI: Body mass index
CAD score: The cardiovascular disease score
GM: Geometric mean
OR: Odds ratio
95% CI: 95% Confidence interval
AR: Androgen receptor
