# Supplementary material for: Association between testosterone and serum soluble α-klotho in U.S. males: a cross-sectional study
Source: BMC Geriatr. 2022 Jul 11;22:570. doi: 10.1186/s12877-022-03265-3 (PMC9275159; doi:10.1186/s12877-022-03265-3)
Supplement: Supplementary file 1 — Additional file 1. Differences of sex hormones and klotho levels among age classificationsa. [file 12877_2022_3265_MOESM1_ESM.docx]

| Tertiles of age | Low | Middle | High | P-value ^b^ |
| --- | --- | --- | --- | --- |
| N | 1204 | 1192 | 1354 | - |
| Age ^c^ | 40-51 | 52-62 | 63-79 | - |
| TT, ng/dl | 396.50±172.24 | 404.73±179.26 | 396.31±199.76 | 0.438 |
| E2, pg/mL | 24.10±9.17 | 24.54±9.80 | 25.00±10.20 | 0.16 |
| SHBG, nmol/L | 38.85±21.01 | 48.23±24.98 | 56.85±26.01 | <0.001 |
| Klotho, pg/ml ^d^ | 800.00  (665.72-968.60) | 796.40  (661.33-974.85) | 765.95  (629.68-948.00) | 0.032 |

Additional Table 1, Differences of sex hormones and klotho levels among age classifications ^a^

^a^ Continuous variables are presented as mean ± standard deviation (SD) and categorical variables are presented as numbers (proportion).

^b^ P values of differences among different TT tertiles were calculated by Kruscal Whallis H test (continuous variables) or chi-square test (categorical variables).

^c^ Ranges of age tertiles were presented as Min-Max.

^d^ S-Klotho concentration is presented in median, upper and lower quartiles due to its highly skew distribution.

Abbr. NHANES, the National Health and Nutrition Examination Survey; TT, total testosterone; E2, estradiol; SHBG, the sex hormone-binding globulin.
