# Supplementary material for: Association between testosterone and serum soluble α-klotho in U.S. males: a cross-sectional study
Source: BMC Geriatr. 2022 Jul 11;22:570. doi: 10.1186/s12877-022-03265-3 (PMC9275159; doi:10.1186/s12877-022-03265-3)
Supplement: Supplementary file 2 — Additional file 2. Subgroup analysis of association between sex hormones and S-Klotho stratified by age among the U.S. males in NHANES 2011-2016a. [file 12877_2022_3265_MOESM2_ESM.docx]

Additional Table 2, Subgroup analysis of association between sex hormones and S-Klotho stratified by age among the U.S. males in NHANES 2011-2016^a^

|  | Younger group (<60) | | |  | Older group (≧60) | | | p for interaction |
| --- | --- | --- | --- | --- | --- | --- | --- | --- |
|  | N^b^ | β, (95% CI) | p value |  | N^b^ | β, (95% CI) | p value |  |
| TT | 2004 | 0.060 (-0.016, 0.137) | 0.123 |  | 1745 | 0.129 (0.057, 0.202) | <0.001 | 0.182 |
| E2 | 1338 | 1.936 (0.305, 3.567) | 0.020 |  | 1188 | 2.551 (0.919, 4.183) | 0.002 | 0.594 |
| SHBG | 1241 | 1.112 (0.375, 1.849) | 0.003 |  | 1115 | 1.733 (1.070, 2.396) | <0.001 | 0.197 |
| T/E2 ratio | 1338 | -82.472  (-832.385,667.442) | 0.829 |  | 1187 | 653.556  (-148.971, 1456.084) | 0.111 | 0.156 |
| TD |  |  |  |  |  |  |  | 0.149 |
| no | 1395 | Ref. | - |  | 1194 | Ref. | - |  |
| yes | 609 | -12.664(-44.252, 18.925) | 0.440 |  | 551 | -29.955(-72.980, 13.070) | 0.185 |  |

^a^ The model was fully adjusted by race, education level, marital status, family income-poverty ratio, BMI, time of venipuncture, CAD score, smoking status, alcohol consumption, and physical activity.

^b^ It was presented with the numbers of observed subjects.

Abbr. NHANES, the National Health and Nutrition Examination Survey; CI, confidence interval; TT, total testosterone; E2, estradiol; SHBG, the sex hormone-binding globulin; T/E2 ratio, the ratio of testosterone to estradiol; TD, testosterone deficiency
