# Supplementary material for: Association between testosterone and serum soluble α-klotho in U.S. males: a cross-sectional study
Source: BMC Geriatr. 2022 Jul 11;22:570. doi: 10.1186/s12877-022-03265-3 (PMC9275159; doi:10.1186/s12877-022-03265-3)
Supplement: Supplementary file 3 — Additional file 3. Subgroup analysis of association between sex hormones and S-Klotho stratified by BMI among the U.S. males in NHANES 2011-2016a. [file 12877_2022_3265_MOESM3_ESM.docx]

Additional Table 3, Subgroup analysis of association between sex hormones and S-Klotho stratified by BMI among the U.S. males in NHANES 2011-2016^a^

|  | Normal weight (BMI≦25) | | |  | Over-weight (BMI 25-30) | | |  | Obese (BMI>30) | | | p for  interaction |
| --- | --- | --- | --- | --- | --- | --- | --- | --- | --- | --- | --- | --- |
|  | N^b^ | β, (95% CI) | p |  | N^b^ | β, (95% CI) | p |  | N^b^ | β, (95% CI) | p |  |
| TT | 855 | 0.079  (-0.016, 0.175) | 0.104 |  | 1562 | 0.105  (0.022, 0.188) | 0.013 |  | 1132 | 0.104  (0.000, 0.207) | 0.050 | 0.909 |
| E2 | 561 | 2.240  (-0.190, 4.669) | 0.071 |  | 1055 | 2.252  (0.354, 4.150) | 0.020 |  | 910 | 2.217  (0.415, 4.018) | 0.016 | 0.999 |
| SHBG | 528 | 0.787  (-0.018, 1.591) | 0.055 |  | 988 | 1.676  (0.858, 2.494) | <0.001 |  | 840 | 2.071  (1.143, 2.998) | <0.001 | 0.088 |
| T/E2 ratio | 561 | 1019.910  (39.575,2000.244) | 0.042 |  | 1055 | -47.781  (-943.402,847.839) | 0.917 |  | 909 | -247.580  (-1352.463,857.303) | 0.661 | 0.158 |
| TD |  |  |  |  |  |  |  |  |  |  |  | 0.787 |
| no | 725 | Ref. | - |  | 1130 | Ref. | - |  | 734 | Ref. | - |  |
| yes | 130 | -29.808  (-106.870, 47.253) | 0.455 |  | 432 | -12.815  (-44.932, 19.302) | 0.441 |  | 598 | -24.926  (-71.883, 22.030) | 0.308 |  |

^a^ The model was fully adjusted by age, race, education level, marital status, family income-poverty ratio, time of venipuncture, CAD score, smoking status, alcohol consumption, and physical activity.

^b^ It was presented with the numbers of observed subjects.

Abbr. BMI, body mass index; NHANES, the National Health and Nutrition Examination Survey; CI, confidence interval; TT, total testosterone; E2, estradiol; SHBG, the sex hormone-binding globulin; T/E2 ratio, the ratio of testosterone to estradiol; TD, testosterone deficiency
