# Supplementary material for: Association between testosterone and serum soluble α-klotho in U.S. males: a cross-sectional study
Source: BMC Geriatr. 2022 Jul 11;22:570. doi: 10.1186/s12877-022-03265-3 (PMC9275159; doi:10.1186/s12877-022-03265-3)
Supplement: Supplementary file 4 — Additional file 4. Subgroup analysis of association between sex hormones and S-Klotho stratified by physical activity among the U.S. males in NHANES 2011-2016a. [file 12877_2022_3265_MOESM4_ESM.docx]

Additional Table 4, Subgroup analysis of association between sex hormones and S-Klotho stratified by physical activity among the U.S. males in NHANES 2011-2016^a^

|  | Less than moderate | | |  | Moderate | | |  | Vigorous | | | p for  interaction |
| --- | --- | --- | --- | --- | --- | --- | --- | --- | --- | --- | --- | --- |
|  | N^b^ | β, (95% CI) | p |  | N^b^ | β, (95% CI) | p |  | N^b^ | β, (95% CI) | p |  |
| TT | 2030 | 0.107  (0.036, 0.178) | 0.003 |  | 342 | 0.121  (-0.074, 0.317) | 0.225 |  | 1377 | 0.077  (-0.008, 0.163) | 0.077 | 0.837 |
| E2 | 1370 | 2.841  (1.386, 4.295) | <0.001 |  | 233 | 5.433  (1.006, 9.860) | 0.016 |  | 923 | 0.328  (-1.729, 2.385) | 0.755 | 0.045 |
| SHBG | 1271 | 1.587  (0.956, 2.218) | <0.001 |  | 216 | 0.741  (-1.040, 2.522) | 0.415 |  | 869 | 1.344  (0.522, 2.166) | 0.001 | 0.634 |
| T/E2 ratio | 1369 | -200.162  (-935.694, 535.370) | 0.594 |  | 233 | -185.092  (-2019.079, 1648.895) | 0.843 |  | 923 | 1075.693  (175.936, 1975.450) | 0.019 | 0.064 |
| TD |  |  |  |  |  |  |  |  |  |  |  | 0.838 |
| no | 1361 | Ref. | - |  | 231 | Ref. | - |  | 997 | Ref. | - |  |
| yes | 669 | -3.617  (-41.959, 34.724) | 0.855 |  | 111 | -34.703  (-101.595, 32.189) | 0.320 |  | 380 | -32.217  (-69.130, 4.695) | 0.099 |  |

^a^ The model was fully adjusted by age, race, education level, marital status, family income-poverty ratio, BMI, time of venipuncture, CAD score, smoking status, and alcohol consumption.

^b^ It was presented with the numbers of observed subjects.

Abbr. NHANES, the National Health and Nutrition Examination Survey; CI, confidence interval; TT, total testosterone; E2, estradiol; SHBG, the sex hormone-binding globulin; T/E2 ratio, the ratio of testosterone to estradiol; TD, testosterone deficiency
